# The bidirectional relationship between subjective visual function and domain-specific cognition in cognitively unimpaired older adults and adults with mild cognitive impairment

**DOI:** 10.3389/fnagi.2024.1465812

**Published:** 2024-11-29

**Authors:** Abigail Dubois, Jordan Sergio, Sima Mozdbar, Ashley Price, Megan Stradtman, Louisa I. Thompson, Peter J. Snyder, Jessica Alber

**Affiliations:** ^1^Interdisciplinary Neuroscience Program, University of Rhode Island, Kingston, RI, United States; ^2^Department of Biomedical and Pharmaceutical Sciences, University of Rhode Island, Kingston, RI, United States; ^3^Burnett School of Medicine, Texas Christian University, Fort Worth, TX, United States; ^4^Butler Hospital Memory and Aging Program, Providence, RI, United States; ^5^Department of Psychiatry and Human Behavior, Alpert Medical School of Brown University, Providence, RI, United States; ^6^George & Anne Ryan Institute for Neuroscience, University of Rhode Island, Kingston, RI, United States

**Keywords:** visual function, aging, domain-specific cognition, mild cognitive impairment, Alzheimer’s disease, preclinical Alzheimer’s disease

## Abstract

**Introduction:**

Subjective visual impairment (VI) is related to cognition in cognitively unimpaired (CU) older adults, mild cognitive impairment (MCI) patients, and Alzheimer’s disease (AD) patients. The utility of subjective VI as an indicator for domain-specific cognitive impairment is unknown.

**Methods:**

We used the National Eye Institute Visual Function Questionnaire (NEI-VFQ-25 item) and a neuropsychological battery to assess the relationship between subjective VI and domain-specific cognitive performance in CU older adults (*N* = 58) and MCI patients (*N* = 16).

**Results:**

The CU group showed a positive relationship between subjective VI and visuospatial performance. CU older adults at high risk for AD demonstrated a unique relationship between subjective VI and attention, processing speed, and executive function. Peripheral vision was related to domain-specific performance in the patient group.

**Discussion:**

Subjective VI complaints may indicate potential for domain-specific cognitive decline in visuospatial performance, executive function, processing speed, and attention in older adults.

## 1 Introduction

Age is one of the leading risk factors for self-reported visual impairment (VI) (Marquié et al., 2019) and the greatest risk factor for Alzheimer’s disease (AD) (Chen et al., 2009). Changes in visual function have been demonstrated early in AD pathogenesis (Bargagli et al., 2020; Wu et al., 2020). However, the direct relationship between VI and cognitive impairment is still under investigation.

VI is known to have a direct impact on quality of life. Previous data shows that those with VI are more likely to report depression in the future, and those with symptoms of depression are more likely to report VI (Horowitz et al., 2005). VI also correlates with AD-associated cognitive decline (Frank et al., 2019; Rashik et al., 2021; Mozdbar et al., 2022). Recent studies have sought to understand the relationship between age-related vision changes and cognitive impairment. One such study by Marquié et al. (2019) reports worsened visual acuity and fewer vision corrections or treatments in older adults with dementia as compared to older adults with either MCI or subjective cognitive decline.

Notably, structural and cellular alterations occur in the eye which reflect AD-related pathology, including lesions of β-amyloid (Aβ) plaques in the lens (Salobrar-García et al., 2019) and thinning of the ganglion cell layer, macular retinal nerve fiber layer (mRNFL) and peripapillary retinal nerve fiber layer (pRNFL) (Snyder et al., 2016; Santos et al., 2018). AD pathology has also been identified within visual pathways. In AD patients, more abundant Aβ plaques and neurofibrillary tangles have been found in the parvocellular layers as compared to magnocellular layers of the lateral geniculate nucleus (LGN) (Marquié et al., 2019). The parvocellular layers of the LGN are primarily for perception of color and fine detail. They are vital for visual acuity (VA) and spatial resolution. A lack of extraocular movement that is linked with a damaged parvocellular pathway could lead to a loss of VA in AD patients as Aβ plaques and neurofibrillary tangles increase with disease progression (Marquié et al., 2019). Comparing self-reported visual functioning and scores on objective cognitive assessments is an important step in understanding the relationship between the experiences of visual and cognitive impairment in preclinical and symptomatic AD.

The relationship between objective visual impairment and cognition is hypothesized to be potentially bidirectional (Vu et al., 2021; Nagarajan et al., 2022). Measures of subjective VI (Nagarajan et al., 2022) and objective VI (Zheng et al., 2018) are strongly related to general cognition in cognitively unimpaired (CU) older adults. Higher scores on subjective VI, as measured using the overall composite score of the National Eye Institute Visual Function Questionnaire (NEI-VFQ-25) (Mangione et al., 2001) were associated with decreased scores on the Mini-Mental State Examination (MMSE), in a sample of CU older adults, MCI patients, and dementia patients in primary care (Mozdbar et al., 2022).

To date, most studies investigating the relationship between VI and cognitive impairment have used the MMSE or the Montreal Cognitive Assessment (MoCA), both of which are cognitive screening tools for primary care, as an outcome measure to assess cognition. What is unknown is whether VI and loss of visual function relates to specific cognitive domains in older adults. Some preliminary work shows relationships between both objective and subjective VI and individual cognitive domains, including: verbal fluency (De La Fuente et al., 2019), executive function (Naël et al., 2019; Varin et al., 2020), visuospatial performance (Naël et al., 2019), attention (Naël et al., 2019; Varin et al., 2020), and processing speed (Naël et al., 2019; Varin et al., 2020). Notably, most work examining the relationship between domain-specific cognition and subjective VI in older adults has a small sample size (*N* < 40) and uses non-standardized self-report to assess subjective VI. Understanding the relationship between VI and domain-specific cognition could provide insight for use in the differential diagnosis of dementias. Early clinical symptoms vary by dementia type. For example, orientation, memory, and word finding are often the earliest clinical manifestations of Alzheimer’s disease, whereas executive function and behavioral change are the earliest manifestations of frontotemporal dementia. Examination of the specific cognitive domains correlated with VI could help to elucidate its potential as a supporting risk/diagnostic biomarker. A recent systematic review (Nagarajan et al., 2022) shows that most studies examine objective visual function in older adults, using measures such as visual acuity and visual field loss. Interestingly, the authors do not differentiate between objective and subjective VI in their systematic review analysis. Examining subjective VI offers a unique insight into visual symptoms and the patient experience. Moreover, previous studies (see Cronin-Golomb et al., 1995; Chen et al., 2017; De La Fuente et al., 2019; Naël et al., 2019; Varin et al., 2020; Lee et al., 2021) examining the relationship between subjective VI and cognition have used primarily non-standardized assessment (i.e., do you feel that you can read and see long distance with your glasses?), rather than reliable and validated measures of subjective VI. Subjective VI has the potential to provide insight into common clinical complaints (i.e., trouble driving at night, difficulty reading signs) and identify whether these complaints relate to specific domains of cognitive impairment.

The aim of this study was to use a valid and reliable measure of subjective VI, the NEI-VFQ, and a neuropsychological battery encompassing tests of executive function, episodic memory, visuospatial construction, language, and attention to understand the relationship between subjective VI and domain-specific cognition in older adults with and without cognitive impairment. The 25-item NEI-VFQ provides thorough insight to visual functioning and a reflection of how visual impairment affects activities of daily living, such as social functioning, mental health, and fulfilling roles (Mangione et al., 2001). Using a domain-specific cognitive battery rather than a cognitive screening tool provides a further and more precise look into how VI relates to different aspects of cognition. Based on previous literature (Zheng et al., 2018; Naël et al., 2019; Varin et al., 2020; Vu et al., 2021; Mozdbar et al., 2022; Nagarajan et al., 2022), we hypothesize that in this population of cognitively unimpaired (CU) older adults and MCI patients, VI will be related to visuospatial construction and attention, processing speed, and executive function.

## 2 Methods

### 2.1 Participants

This study used data collected from 74 participants in the Atlas of Retinal Imaging in Alzheimer’s Study (ARIAS) (Alber et al., 2020), aged 55–80. Inclusion criteria have been published previously (Alber et al., 2020). In brief, inclusion criteria included adequate visual and auditory acuity allowing for neuropsychological testing, ability to provide written informed consent, medications stable for 4 weeks prior to screening, and having a study partner to participate in study visits (to provide informant ratings on functional measures). Exclusion criteria included a history of other ocular or neurological disease, history of severe brain injury or other known neurologic disease, history of stroke with lasting impairment to the visual system, a current diagnosis of epilepsy, Geriatric Depression Scale score > 6, poorly controlled psychiatric disorder, history of alcohol or substance abuse, history of schizophrenia or psychotic features, any unstable medical condition leading to difficulty to comply with protocol, history of systemic cancer within the past 5 years, history of clinically significant liver disease, history of myocardial infarction, uncontrolled hypertension, uncontrolled or insulin requiring diabetes, history of narrow-angle glaucoma, and/or history of elevated intraocular pressure. Additionally, participants with dementia were excluded from this study. Medical history was collected from all participants and their informant/study partner at a screening visit to assess inclusion/exclusion criteria. All participants provided written informed consent in accordance with the Declaration of Helsinki.

In ARIAS, participants were divided into three groups based on cognitive status and apolipoprotein (APOE) genotype: (1) cognitively unimpaired—low risk (Montreal Cognitive Assessment (MoCA) > =26, Clinical Dementia Rating (CDR) = 0, Repeatable Battery for the Assessment of Neuropsychological Status Update (RBANS) Delayed Memory Index (DMI) >85, APOE E4 allele non-carrier, no first degree family history of AD, *N* = 40); (2) cognitively unimpaired—high risk (MoCA ≥ 26, CDR Score = 0, RBANS DMI > 85, APOE E4 allele carrier, positive first degree family history of AD, *N* = 18); (3) patient group: MCI (MoCA > 19 and <24 at screening, CDR = 0.5, ≤85 on RBANS DMI, *N* = 16). The MoCA is a commonly used screening tool deployed often in primary care. It is scored out of 30 possible points, with scores ≥26 indicating that someone is cognitively unimpaired. Scores below 26 are indicative of necessary follow-up evaluation for cognitive impairment (Nasreddine et al., 2005). The CDR is a structured interview assessment used by clinicians to assess functional impairment in older adults. It consists of both an informant/study partner interview and a patient interview, and in this study CDR’s were completed by board-certified neuropsychologists to assess functional impairment. The CU group had CDR global scores of 0, while the MCI group had CDR global scores of 0.5, which is associated with mild cognitive impairment (Morris, 1997). APOE E4 is a risk gene for AD. While multiple APOE variants exist (E2, E3, and E4), possessing one copy of the E4 allele confers a 25–30% risk of cognitive decline with onset prior to age 85, whereas possessing two copies confers a 51–60% risk (Genin et al., 2011). The E4 allele is common among the general population with 25% of people possessing at least one copy. Specifically, among patients with AD, the E4 allele is more common with 40% of patients possessing at least one copy (Genin et al., 2011).

### 2.2 Procedures

The NEI-VFQ-25 (Mangione et al., 2001) was administered at baseline to assess subjective VI. This is a self-report questionnaire that uses 12 subscales measuring different domains of visual health including: general health, general vision, near activities, distance activities, driving, peripheral vision, color vision, ocular pain, and vision-related role difficulty, dependency, social function, and mental health. Each subscale was scored on a scale of 0–100 with 100 being the best possible score. The scores of each subscale, excluding general health, were computed as an unweighted average to find an overall composite score. The NEI-VFQ-25 is a reliable tool that reproduces valid data across numerous conditions and settings (Mangione et al., 2001).

Daily functioning was assessed using the CDR (Morris, 1997). General cognition was assessed using MoCA (Nasreddine et al., 2005) and the RBANS-U (Duff et al., 2008; Karantzoulis et al., 2013) total score. Episodic memory was assessed using the RBANS Delayed Memory Index (RBANS DMI), and attention, executive function, and processing speed were assessed using the digit-symbol substitution test (DSST) (Wechsler, 2008). Of note is that the DSST involves aggregation of all three of these cognitive domains, and performance is reflective of this rather than of the domains in isolation. Notably, this assessment has been used in studies examining the relationship between both objective and subjective VI and cognition (Chen et al., 2017; Swenor et al., 2019). Cognitive data was collected at participant screening (CDR, MoCA, RBANS) for group assignment, and baseline visits (NEI-VFQ, DSST), which were completed within a six-week period. Normed scores were used for analysis of RBANS-U and DSST scores.

### 2.3 Statistical analysis

Statistical analyses were conducted using RStudio (version 4.3.1, Vienna, Austria). ANOVAs and chi-squared analyses, as applicable, were used to compare demographic factors between the low risk, high risk, and patient groups. For correlation analyses, normality and outliers were assessed using QQ-plots and boxplots, respectively. For regression analyses, normality was assessed using QQ-plots and variables were transformed using the appropriate transformation (i.e., squared transformation) when this assumption was violated. Outliers were assessed using Cook’s Distance. Conventional cook’s distance cutoffs of 4/n or 1 are either too strict or too conservative, respectively, and therefore we used a cutoff of 0.5. Pearson correlation was used to examine the association between the NEI-VFQ composite scores and scores on each cognitive assessment. Finally, linear regression controlling for age, years of education, and sex was used to examine: (1) whether subjective visual impairment on the NEI-VFQ predicted cognitive test results of all significant correlations; and (2) whether domain-specific cognitive performance predicted subjective visual impairment (NEI-VFQ composite score).

## 3 Results

### 3.1 Demographics

A total of 74 participants completed the screening and baseline cognitive measures and NEI-VFQ, 40 in the CU low risk group, 18 in the CU high risk group, and 16 in the patient group. The mean age of the sample was 66.69 years, and the sample was 51% female, with most CU participants identifying as female, and much of the patient group identifying as male (see Table 1). As expected, the patient group had a significantly lower MoCA score than the CU high risk and CU low risk groups (*p* < 0.01, see Table 1). The CU low risk group (APOE E4 non-carriers) had 0 APOE E4 alleles, and the CU high risk group all carried at least one APOE E4 allele. In the patient group, 50% (*N* = 8) of the patients carried at least one APOE E4 allele. There were no other significant group differences between groups on demographic variables (see Table 1).

**Table 1:** Participant demographics for the entire sample (*N* = 74), broken down by participant group.

	Total sample (*N* = 74)	CU low risk (*N* = 40)	CU high risk (*N* = 18)	Patient group (*N* = 16)	*p*-value
Age	66.69 (6.04)	66.60 (6.32)	64.50 (5.85)	69.37 (4.77)	0.06
Education	16.00 (2.37)	16.05 (1.89)	15.83 (2.95)	16.33 (2.71)	0.94
Sex (% female)	51	50	72	31	0.06
MoCA Score	26.53 (2.77)	27.30 (1.62)	27.94 (1.26)^^^	23.00 (3.00)^*^	<0.01
APOE genotype (% E4 carriers)	35%	0%	100%	50%^*^	<0.01

* Significantly different from the CU (low risk) and CU (high risk) groups.

^ Significantly different from the CU (low risk) group.

CU, cognitively unimpaired; MoCA, Montreal Cognitive Assessment; APOE, apolipoprotein E.

### 3.2 NEI-VFQ composite scores and cognition

#### 3.2.1 Entire sample (*N* = 74)

We ran Pearson’s correlation tests across the entire sample to determine associations between subjective VI and domain-specific cognition. The Holm multiple comparisons test was used to correct for type I error. There was a significant association between scores on DSST and the composite score of the NEI-VFQ (*r* = 0.280, *p* = 0.017), as well as a trend level correlation between the visuospatial subscale of the RBANS and the NEI-VFQ composite score (*r* = 0.226, *p* = 0.054, see Table 2).

**Table 2:** Pearson’s correlations between the NEI-VFQ composite score and the RBANS Visuospatial Scale (RBANS VS), the RBANS Delayed Memory Index (RBANS DMI), and the Digit Symbol Substitution Task (DSST).

Sample	Cognitive test	Pearson’s correlation (*r*)	*p*-value
Entire sample (*N* = 74)	RBANS VS	0.226	0.05
	RBANS DMI	−0.127	0.29
	DSST	0.280	**0.02**
Total CU (*N* = 58)	RBANS VS	0.334	**0.01**
	RBANS DMI	−0.099	0.47
	DSST	0.325	**0.01**
Low risk CU (*N* = 40)	RBANS VS	0.294	0.06
	RBANS DMI	0.088	0.60
	DSST	0.242	0.13
High risk CU (*N* = 18)	RBANS VS	0.431	0.07
	RBANS DMI	−0.473	**0.05**
	DSST	0.496	**0.04**
MCI group (*N* = 16)	RBANS VS	−0.116	0.68
	RBANS DMI	−0.394	0.146
	DSST	−0.083	0.777

CU, Cognitively Unimpaired; RBANS, Repeatable Battery for the Assessment of Neuropsychological Status; VS, Visuospatial sub-score; DMI, Delayed Memory Index; DSST, Digit Span Substitution Test. Bold values means statistically significant (*p*<0.05).

#### 3.2.2 Participant groups

While there were no significant associations between visual function and any cognitive domains in the patient group, there was a significant association between the NEI-VFQ composite score and both the visuospatial subscale of the RBANS (*r* = 0.334, *p* = 0.011) and the DSST (*r* = 0.325, *p* = 0.013) across all CU participants (see Table 2).

**Figure 1:**
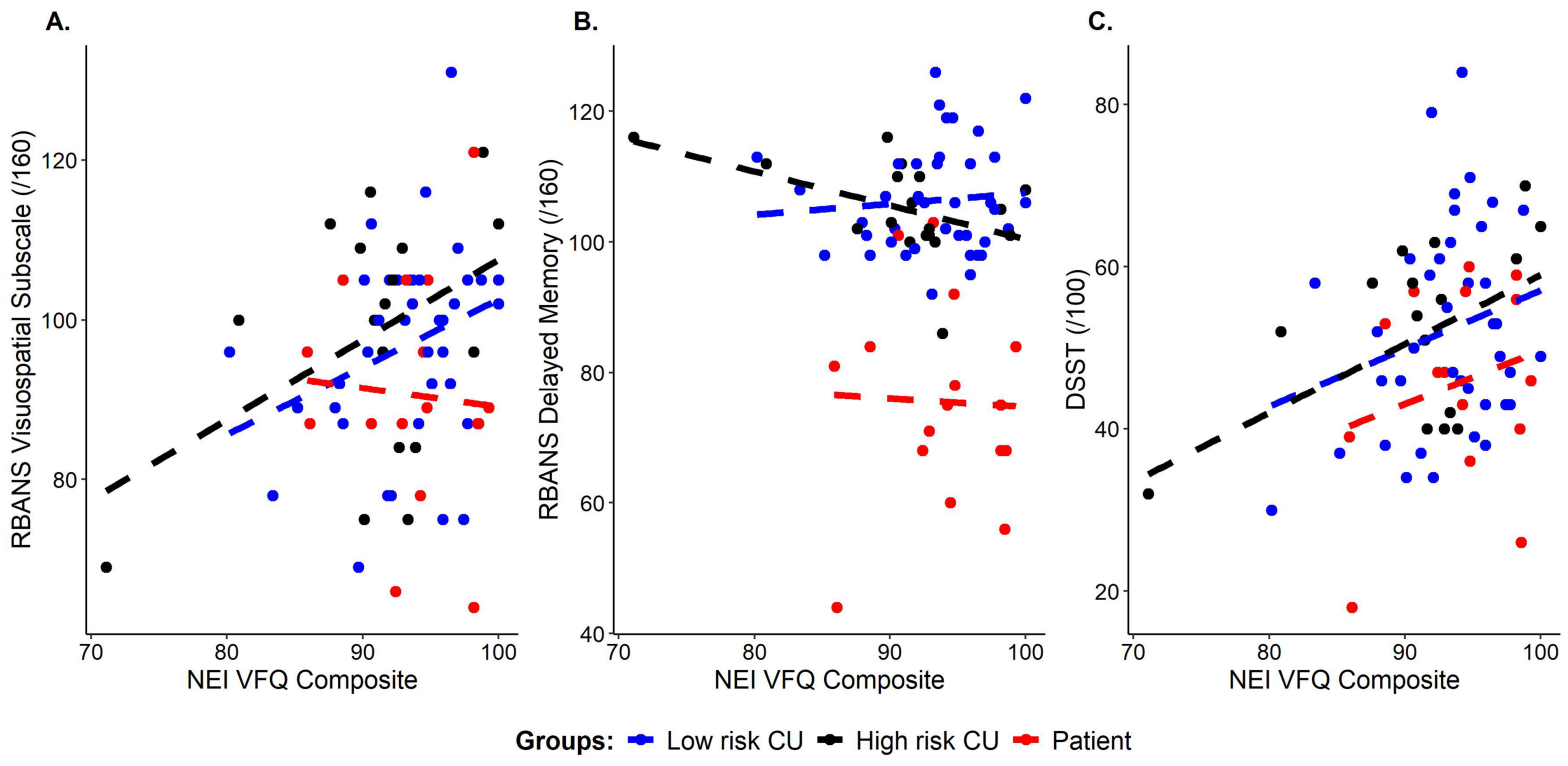
Correlations between subjective visual functioning (NEI-VFQ composite scores) and cognitive performance on: **(A)** visuospatial ability (RBANS visuospatial subscale score), **(B)** episodic memory (RBANS delayed memory index score), and **(C)** processing speed, executive function, and attention (DSST total score), color coded by group. Blue = cognitively unimpaired low risk, Black = cognitively unimpaired, high risk, Red = patient group.

Broken down by AD risk, we found a trend level correlation between the visuospatial subscale of the RBANS and the NEI-VFQ composite score in both the low-risk CU (*r* = 0.294, *p* = 0.069) and high-risk CU (*r* = 0.431, *p* = 0.074) groups. Interestingly, there were also significant associations between the NEI-VFQ composite score and both the RBANS delayed memory subscale (*r* = −0.473, *p* = 0.047) and DSST (*r* = 0.496, *p* = 0.036) in the high-risk group only (see Figure 1; Table 2).

### 3.3 NEI-VFQ composite score may predict delayed episodic memory performance

**Figure 2:**
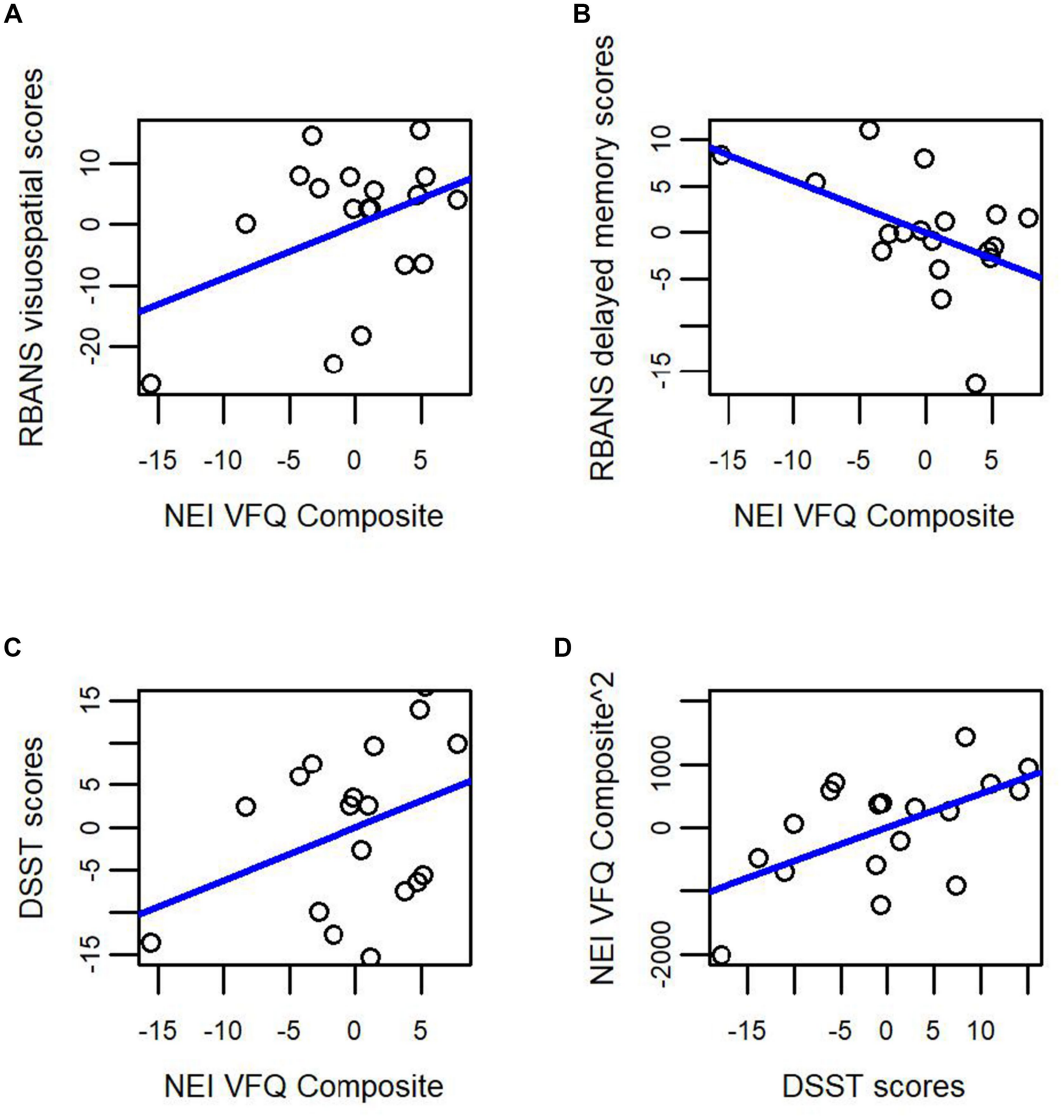
Subjective visual impairment (NEI-VFQ composite score) as a predictor of domain-specific cognitive impairment in cognitively unimpaired older adults at high risk for Alzheimer’s disease. Subjective visual impairment as measured by the NEI VFQ composite score does not significantly predict **(A)** visuospatial performance (RBANS visuospatial index score); or **(C)** attention, executive function, and processing speed (DSST score). Subjective visual impairment as measured by the NEI-VFQ composite score approached statistical significance as a predictor of **(B)** episodic memory performance (RBANS delayed memory index). Finally, in **(D)** performance on the DSST, a complicated task of attention, processing speed, and executive function, predicted subjective visual impairment (NEI-VFQ composite score) in cognitively unimpaired older adults at high risk for Alzheimer’s disease.

We used linear regression to determine whether subjective VI (as measured by the NEI-VFQ composite score) predicted domain-specific cognition on the RBANS DMI, RBANS visuospatial scale, and DSST. We ran three regression models examining whether the overall composite score of the NEI-VFQ predicted the outcome variable (RBANS DMI, RBANS visuospatial scale, or DSST), while controlling for age, years of education, and sex. In the model with RBANS visuospatial score as the outcome measure, age was the only significant predictor (*β* = −0.154, *p* < 0.05, adjusted *R*^2^ = 0.345, Figure 2A). The NEI-VFQ composite score approached statistical significance as a predictor of RBANS DMI score (*β* = −0.557, *p* = 0.055, adjusted *R*^2^ = 0.281, Figures 1B, 2B), while none of the covariates (age, sex, education) were significant predictors of RBANS DMI score. In the model with DSST as the outcome variable, the covariates (age, sex, education) and the NEI-VFQ composite scores were not significant predictors of performance on the DSST (adjusted *R*^2^ = 0.154, Figure 2C).

### 3.4 DSST performance predicts subjective visual important only in cognitively unimpaired older adults at high risk for Alzheimer’s disease

While accounting for age, sex and years of education, our overall regression model revealed that 51% of the proportion of variability in NEI-VFQ composite score was accounted for by our predictors (adjusted *R*^2^ = 0.508, *F* (4, 3) = 5.384, *p* = 0.009). The DSST (*β* = 52.72, *p* = 0.022) significantly predicted scores on the NEI-VFQ composite (see Figure 2D).

## 4 Discussion

The purpose of this study was to investigate the relationship between subjective visual impairment and domain-specific cognitive performance using a valid and reliable measure of subjective VI, the NEI-VFQ, and a domain-specific neuropsychological battery. Since visual impairment is an early symptom in Alzheimer’s disease, the subjective experience of visual impairment may be a predictor of cognitive performance in older adults in preclinical and symptomatic stages of AD.

We found an positive association between subjective VI and executive function, attention, and visuospatial ability in CU older adults. When breaking down participants by AD risk group, we saw a unique profile in the high-risk CU older adults where the NEI-VFQ composite score was related to performance on the DSST, a test of attention, executive function, and processing speed. In this group, subjective VI predicted episodic memory performance on the RBANS at a trend level, and performance on tasks of episodic memory, attention, processing speed, and executive function predicted subjective VI, indicating a bi-directional relationship between subjective VI and cognition. To our knowledge, this is the first study to examine this relationship in CU older adults. A systematic review and meta-analysis showed that older adults with *objective* VI were more likely to have cognitive impairment and dementia; but the inverse relationship (those with cognitive impairment having objective VI) was more tenuous (Vu et al., 2021).

There were no significant relationships found between subjective VI and cognition in the patient group, which was unexpected. Previous work (Mozdbar et al., 2022) found that general cognition measured by the MMSE was associated with decreasing scores on the NEI-VFQ composite in a sample of CU (*N* = 80), MCI (*N* = 35) and dementia (*N* = 16) participants. There were no subgroup analyses performed; it’s possible that this relationship was driven by the CU group. An alternative explanation is a lack of reliable self-report data on the NEI-VFQ in our patient group due to lack of insight or denial of symptoms. It is possible that subjective VI changes affecting domain-specific cognition peak in the preclinical stage of AD dementia, rather than the symptomatic stages. This is supported by our observation of a unique relationship between subjective VI and attention, processing speed, and executive function in our CU, high risk group. Future research examining subjective VI and domain-specific cognition in patient groups should include longitudinal observation of larger samples to determine whether the relationship between subjective VI and cognition remains in AD. We did find that the peripheral vision subscale on the NEI-VFQ showed a relationship to general cognition and performance on the DSST, a task of attention, processing speed, and executive function in the patient group. Previous research has shown a relationship between retinal ganglion cell loss leading to loss of peripheral vision and risk of driving accidents in older adults and MCI/dementia patients (Henderson and Donderi, 2004; Chen et al., 2017; Anderson et al., 2021). Although we did not assess driving safety in this study, these findings in our entire sample/patient population align with previous data demonstrating peripheral vision and peripheral motion sensitivity are affected by natural aging and neurodegenerative disease (Chen et al., 2017).

We found an expected correlation between visuospatial performance and subjective VI in our cognitively unimpaired sample. Our finding that performance on the DSST, a task that relies heavily on visual input in copying symbols, predicted subjective VI, is somewhat intuitive. The finding that this is unique to CU older adults at high risk for AD supports the hypothesis that visual changes early in the AD pathophysiologic cascade can predict cognitive performance in this population. This finding is consistent with larger findings in two population-based samples of CU older adults (Chen et al., 2017), which found that subjective VI predicted reduced performance on the DSST and decline in cognitive performance over a 10-year follow-up period.

Our findings with respect to episodic memory in the high-risk CU group were paradoxical; as subjective VI increased, episodic memory performance improved. One potential reason for this is that there was a single participant who had comparatively low scores on subjective VI (composite score = 71/100), but an RBANS DMI of 120. Upon further examination, this individual had a lower NEI-VFQ composite score due to difficulties on the near vision sub-scale. It is very likely that this finding in the high-risk cohort is driven by this single observation, and therefore we cannot draw the conclusion VI and episodic memory are related in CU high risk older adults. Indeed, when we repeated our analysis without this observation, the relationship between subjective VI and episodic memory was not significant.

Limitations of this study include small sample sizes for the CU high risk group. Larger samples are required to replicate the unique findings of deficits in attention, processing speed, and executive function in adults at high risk for AD development. Additionally, future research may further examine cognitive performance and subjective VI among CU older adults with and without biomarker confirmed preclinical AD. We did not have biomarker confirmation in this sample, but instead used APOE E4, subjective cognitive complaints, and first-degree family history to assess AD risk level in CU older adults. Furthermore, while we separated cognitively unimpaired based on AD risk, some of these older adults may go on to develop other types of dementia, such as progressive supranuclear palsy (PSP) and dementia with Lewy bodies (DLB), which have domain-specific cognitive profiles and known effects on the visual system.

Overall, these data support the hypothesis that subjective VI may be an indicator of domain specific cognitive performance in CU older adults at high risk for AD. Longitudinal studies in larger samples are ongoing to examine whether subjective VI reliably predicts cognitive decline in high-risk CU older adults. The current findings provide insight into the relationship between subjective VI and cognition in older adults and suggest that reports of VI should be noted as a concern in this population. When considered in combination with known AD risk factors, a more comprehensive evaluation may be warranted. This may lead to improvements in health-related quality of life for these patients and positively affect cognitive outcomes.

## Data Availability

The raw data supporting the conclusions of this article will be made available by the authors without undue reservation.
